# Our January (1886) Number

**Published:** 1885-12-20

**Authors:** 


OUR JANUARY (1886) NUMBER will contain a useful condensed exhibit of Weights and Measures, both of the ordinary weight and the metric system ; also of the Thermometric scales.

---

*Leonard's Physician's Pocket Day-book;* bound in red morocco, with flap, pocket, and pencil loops. Price, postpaid, $1. Published annually by the Illustrated Medical Journal, Detroit, Mich.

The above is an excellent pocket day-book, containing day charges for twenty-five patients.

